# Network tuned multiple rank aggregation and applications to gene ranking

**DOI:** 10.1186/1471-2105-16-S1-S6

**Published:** 2015-01-21

**Authors:** Wenhui Wang, Xianghong Jasmine Zhou, Zhenqiu Liu, Fengzhu Sun

**Affiliations:** 1 Molecular and Computational Biology Program, University of Southern California, 1050 Childs Way, Los Angeles, USA; 2 Samuel Oschin Comprehensive Cancer Institute, Cedars-Sinai Medical Center, 8700 Beverly Blvd., Los Angeles, USA; 3 Centre for Computational Systems Biology, School of Mathematical Sciences, Fudan University, 220 Handan Rd, Shanghai, PR China

**Keywords:** Rank Aggregation, Network, CGI, Gene Rank, Endeavour, RRA

## Abstract

With the development of various high throughput technologies and analysis methods, researchers can study different aspects of a biological phenomenon simultaneously or one aspect repeatedly with different experimental techniques and analysis methods. The output from each study is a rank list of components of interest. Aggregation of the rank lists of components, such as proteins, genes and single nucleotide variants (SNV), produced by these experiments has been proven to be helpful in both filtering the noise and bringing forth a more complete understanding of the biological problems. Current available rank aggregation methods do not consider the network information that has been observed to provide vital contributions in many data integration studies. We developed network tuned rank aggregation methods incorporating network information and demonstrated its superior performance over aggregation methods without network information.

The methods are tested on predicting the Gene Ontology function of yeast proteins. We validate the methods using combinations of three gene expression data sets and three protein interaction networks as well as an integrated network by combining the three networks. Results show that the aggregated rank lists are more meaningful if protein interaction network is incorporated. Among the methods compared, CGI_RRA and CGI_Endeavour, which integrate rank lists with networks using CGI [1] followed by rank aggregation using either robust rank aggregation (RRA) [2] or Endeavour [3] perform the best. Finally, we use the methods to locate target genes of transcription factors.

## Introduction

As new biotechnologies, such as microarray, genotyping, and next generation sequencing (NGS), continue to be developed and the cost of biological experiments decreases, a biological phenomenon can be studied using different technologies or by different research groups. Each study can yield a rank list of genes according to the strength of association of genes with the biological phenomenon of interest. These studies can include the detection of differentially expressed genes under certain perturbations, disease status or treatments; association of single nucleotide variants (SNV), copy number variation (CNV), methylation, and alternative splicing with phenotypes; or identification of target genes of transcription factors or other regulatory molecules including various RNAs. For example, in the study of associating SNVs to a disease, SNVs can be ordered using the p-values based on the χ^2 ^test for equal allele frequency between the cases and controls. Similarly, in the study of differentially expressed genes for a disease status, genes can be ranked by comparing the genes' expression levels between cases and controls. Regulatory targets of a transcription factor can be ranked according to the differential expression levels of genes from knock out experiments of the transcription factor.

In order to have a more complete understanding of the biological phenomena of interest taking into account the multiple studies rather than using individual ones, efficient aggregation of the results from the individual studies is needed. Since these studies can use different technologies and are generally carried out from different laboratories, global normalization of the data across these studies can be challenging. Thus, aggregation of the rank lists of the genes provides a promising approach for integrating the results from these studies allowing scientists to better interpret experimental results, understand the potential mechanism and determine following up experiments [4-6]. The inputs of rank aggregation are gene (or SNP, domain, protein, pathway) rank lists and the output is a combined rank list that is anticipated to be more meaningful than any single rank list.

Many methods have been developed for rank list aggregation using the same type of data such as gene expression profiles, which we refer as horizontal data integration. Manor *et al*. [7] proposed a method to rank the risk of SNVs causing disease, in which the data is re-sampled multiple times and a SNV ranking is generated for each such sample, with the final SNV ranking being an aggregation of rankings from all the samples. Aerts *et al*. [3] integrated disease gene rank lists from multiple data sources using order statistics [8]. Alder *et al*. [9] used rank aggregation method to merge information from different microarray data sets into a single global rank list to show the expression level similarity. Kolde *et al*. [2] developed a new rank aggregation method intended to improve the order statistic from [3]. Jiang *et al*. [10] used a similar rank aggregation method as in [3] to integrate multiple information sources to rank causal genes for livestock diseases. Sun *et al*. [11] developed a software package for integrating rank lists from pathway enrichment analysis of different data sets, such as transcriptomics, proteomics, metabolomics and genome wide association studies (GWAS). Trepper *et al*. [12] found genes showing mRNA-level response to changes in DAF-16 activity for further experimental validation by integrating publicly available data from genome wide association studies with a rank integration method. Lin [13] reviewed the available rank aggregation methods at that time and classified them into three categories. The first category is based on Thurstone's model [14] that is originally designed for marketing and advertisement research. The second category is based on heuristic algorithms including Borda's method [15] and Markov chain based methods [16]. The third category is based on stochastic optimization minimizing the average distance between the input rank lists and the aggregated rank list [17]. In a more recent study, Deng *et al*. [18] developed a Bayesian approach for rank aggregation that can estimate the reliability of different rank lists and use the reliability to weight the different lists.

Another type of integration is to combine different types of data such as gene expression profiles and molecular networks, which we refer as vertical data integration. Realizing that genes that are in the same pathway or close together in networks are more likely to perform similar functions and thus have similar ranks, investigators have developed a variety of different methods to integrate different types of data such as gene expression and networks [19-21] or association results of genes with molecular networks [22-24]. Wang *et al*. [21] proposed a network guided gene ranking method by firstly utilizing networks to find coordinative components representing the underlying biological processes or pathways and then projecting gene expression data onto the coordinative components to estimate the association strengths of genes to a biological phenomenon. These association strengths are then used to rank the genes. Lavi *et al*. [20] introduced a kernel based on the network topology and then used support vector machine to detect disease biomarkers from gene expression data. Garcia *et al*. [19] proposed an approach to find the sub-network component associated with extreme values of a list of genes. Hofree *et al*. [22] developed a method to stratify the different cancer types into informative subtypes by clustering together patients with mutations in similar network regions based on integration of somatic mutation profiles and networks. Novarino *et al*. [24] studied the genetic basis of hereditary spastic paraplegias (HSP) by using whole-exome sequencing in combination with network analysis. They identified 18 previously unknown putative HSP genes and generated a host of other candidate genes for future study. Jia and Zhao [23] put forward a cancer driver gene prediction method by firstly identifying the significantly mutated genes with generalized additive models based on sample-specific mutation profiles and then collected the novel interaction neighbors of the mutant genes in the network with the help of random walk with restart. Mutant genes and the corresponding interaction neighbors for each sample are heuristically integrated to present the final prediction result.

Despite the many studies on either horizontal or vertical data integration, no studies are available to consider both multiple rank lists and networks simultaneously. In this paper, we study whether we can improve the performance of rank list aggregation by incorporating network information. Our basic assumption is that genes that are close together in networks have similar ranks in the list. Therefore, we firstly tune each given rank list with network and then aggregate the updated rank lists. "Combining Gene expression with Interaction (CGI)" [1] and "GeneRank (GR)" [25] are two widely recognized methods for integrating network information with gene expression data. We modify these methods so that they can be applicable to rank lists. Endeavour [3] and RRA [2] are then used to aggregate the rank lists leveraged by the network. We study the effectiveness of incorporating networks in rank list aggregation by comparing the performance of all the combinations, CGI_Endeavour, CGI_RRA, GR_Endeavour, and GR_RRA using three yeast gene expression data sets and three protein interaction networks as well as the integrated network. We found that by incorporating network information, the performance of rank list aggregation is improved significantly in most cases. Among these methods, CGI_RRA and CGI_Endeavour perform the best.

Focusing on CGI_RRA and CGI_Endeavour, we show that the improvement really originates from the network information by implementing the methods on network with shuffled labels. By running the methods on networks with noisy interactions, we show the performance of CGI_RRA and CGI_Endeavour decreases as the noise level increases as expected, but is relatively robust to noise when the noise level is as high as 40%. At last, we show the applicability of our methods on predicting pathway members.

## Materials and methods

In this section, we present methods to integrate multiple rank lists with network information for gene prioritization. For each rank list, we first modify two methods, Combining Gene expression and protein Interaction (CGI) [1] and GeneRank (GR) [25], for improving the gene rank by integrating the original rank list with the protein interaction network. We then aggregate the updated rank lists using Endeavour [3] and Robust Rank Aggregation (RRA) [2].

Assuming that there are *m *rank lists of *n *genes, *r*_*j *_= (*r*_1,*j*_, *r*_2,*j*_,⋯,*r_n_*,_*j*_)', *j *= 1,⋯, *m*, where *r_i,j _*is the rank of the *i*-th gene in the *j*-th list. First, each rank list is normalized into rank ratio list, *ra_i,j _*= *r_i,j_*/*_n_*, *i = *1,⋯, *n*. Second, rank ratio lists are transformed to score lists based on the inverse of standard normal distribution function.

(1) $$F\left(x\right)=\int_{-\infty }^{x}e^{-\frac{s^{2}}{2}}ds,\;\;\;z_{i,j}=-F^{-1}\left(ra_{i,j}\right).$$

If *ra_i,j _*= 1, it is substituted by *ra_i,j _*= 0.9999 so that the value of *z_i,j _*is well defined. The value of *z_i,j _*ranges in (-∞, +∞) and high value of *z_i,j _*corresponds to high rank. This transformation makes the distribution of *z_i,j _*for fixed *j *to be standard normal so that the integration with the network is more stable.

In addition to a set of rank lists, we also assume that there is a protein interaction network. Let *H *denote the adjacency matrix of the input network where the nodes indicate proteins and the edges indicate interactions. If two nodes *u *and *v *are connected in the network, *H_u,v _*= 1; Otherwise, *H_u,v _*= 0.

### Update rank lists using CGI

Ma *et al*. [1] developed a method, CGI, to integrate gene expression profiles with protein interaction network for gene prioritization. The basic idea is that if a gene and most of its neighbors are associated with a phenotype of interest, the gene is more likely to be true causal genes for the phenotype compared to another gene with non-associated neighbor genes. Therefore, Ma et al. [1] proposed to use the weighted average association strength of the gene and its neighbors with the phenotype to measure the likelihood that the gene is related to the phenotype. Close neighbors are weighted more heavily than distant neighbors. One key question is how to define closeness between nodes in the network. The authors showed that diffusion kernel defined in [26] performed well compared to direct neighbors or shortest distance on networks for gene prioritization.

In this study, we extend CGI so that it can be applied to rank based data. CGI first needs to define a similarity measure between two genes based on the protein interaction network. Many different similarity measures can be defined including direct neighbors, shortest distance, and diffusion kernels as reviewed recently in [27]. Previous studies have shown the superiority of diffusion kernel similarity measure over direct neighbor and shortest distance similarity measures. Therefore, we concentrate on the use of diffusion kernel in this study. The diffusion kernel is defined as *S *= *e*^-*τL*^, where r is a tuning parameter and *L *= *D *- *H *is the Laplacian matrix of *H*, where *D *is a diagonal matrix with the diagonal elements containing the node degrees. The elements in score matrix *S *is normalized as $K_{u,v}=S_{u,v}\text{/}\sqrt{S_{u,u}S_{v,v}}$. The diffusion kernel represents a global similarity between nodes in a network, with higher values representing closer relationship. CGI corresponding to CGI_3 _in [1] is defined as

(2) $$R_{i,j}=\frac{z_{i,j}+\lambda \sum_{l=1,l\neq i}^{n}|z_{l,j}|K_{l,i}}{1+\lambda \sum_{l=1,l\neq i}^{n}K_{l,i}},\;\;i=1,2,\cdots \;,n,$$

where λ is a tuning parameter that controls the contribution of the neighbor genes and *z_i,j _*is defined in equation 1. The updated value of *R_i,j _*indicates the strength of association of gene *i *with the phenotype in the *j-*th list. Large value of *R_i,j _*corresponds to high rank.

### Update rank lists using GR

GeneRank (GR) [25] is a widely used method for gene prioritization by integrating gene expression profiles with a network. GR is based on similar ideas as PageRank originally designed for ranking web pages and was extended to gene expression analysis in [25]. Let *c_i _*be the absolute value of the correlation coefficient between the *i*-th gene expression levels with the phenotype, $r_{i}^{\left(0\right)}=c_{i}\text{/}\sum_{i}c_{i}$, and $r^{\left(0\right)}=\left(r_{1}^{\left(0\right)},r_{2}^{\left(0\right)},\cdots \;,r_{n}^{\left(0\right)}\right)\prime$. The ranking of the genes is based on the solution to

$$\left(I-dH\;D^{-1}\right)X=\left(I-d\right)r^{\left(0\right)}.$$

Here we adapt the method for integrating gene ranks with a network. For the above transformed *z *score, we further transform it following the procedures for GR. For the *i*-th element in the *j-*th rank list, let $R_{i,j}^{\left(0\right)}=z_{i,j}\text{/}||z_{j}|{|}_{1}$, where $\left||z_{j}|{|}_{1}=\sum_{i}|z_{i,j}\right|$. For the *j-*th rank list, let

$$R_{j}^{\left(k\right)}=\left(1-d\right)z_{j}+dHD^{-1}R_{j}^{\left(k-1\right)},\;\;k=1,2,\cdots \;,$$

where *d *is a tuning parameter, *z_j _*is a column vector with the *i*-th element as *z_i,j _*and $R_{j}^{\left(k\right)}$ is a column vector with the *i*-th element as $R_{ij}^{\left(k\right)}$. If *d *is small, more weight is assigned to the original score. While if *d *is large, more weight is given to the neighboring genes and network information will play a more important role. When $R_{j}^{\left(k\right)}$ finally stabilized (i.e $\underset{i}{\text{max}}\text{|}R_{ij}^{\left(k\right)}-R_{ij}^{\left(k-1\right)}|<1\times 10^{-6}$) or the number of iterations reaches a large number (1000 in this paper), the iteration stops. The limit of $R_{j}^{\left(k\right)}$ as *k *tends to infinity denoted as *R_j _*satisfies the equation

$$\left(I-dHD^{-1}\right)R_{j}=\left(1-d\right)z_{j},\;\;j=1,2,\cdots \;,m.$$

We use the component values of *R_j _*to rank the genes with larger values ranked higher for the *j-*th list.

### Aggregate rank lists using Endeavour

The component scores of *R_j _*from CGI and GR can be used to re-rank the genes. A new set of rank lists $r_{j}^{new},j=1,\cdots \;,m$, incorporating network information are produced, which are then aggregated in the second step. Let $r_{i,j}^{new}$ be the rank of the *i*-th gene in the *j-*th list. Stuart *et al*. [8] proposed a rank list aggregation statistic calculated from all rank ratios using the joint cumulative distribution of a multidimensional order statistic. Assuming $r_{\left(i\right)}^{new}=\left\{r_{i,1}^{new},\cdots \;,r_{i,m}^{new}\right\}$ is ordered rank ratio vector for gene *i *across *m *rank lists in ascending order. Let

$$Q\left(r_{\left(i\right)}^{new}\right)=m!\int_{0}^{r_{i,1}^{new}}\int_{s_{1}}^{r_{i,2}^{new}}\cdots \int_{s_{m-1}}^{r_{i,m}^{bew}}d_{s_{m}}\cdots d_{s_{2}}d_{s_{1}}.$$

Aerts *et al*. [3] developed an efficient method to calculate the statistic using the iteration $V_{k}=\sum_{l=1}^{k}{\left(-1\right)}^{l-1}\frac{V_{k-l}}{l!}r_{i,m-k+1}^{new},Q\left(r_{\left(i\right)}^{new}\right)=m!V_{m},\;\;V_{0}=1$. This iteration method is used in our paper to calculate $Q\left(r_{\left(i\right)}^{new}\right)$, which are then used to rank genes with smaller values of $Q\left(r_{\left(i\right)}^{new}\right)$ ranked higher.

### Aggregate rank lists using RRA

Kolde *et al*. [2] proposed another rank aggregation method that assumes the null distribution of all the rank ratios as uniform on the unit interval. The same rank ratio vector $r_{\left(i\right)}^{new}=\left\{r_{i,1}^{new},\cdots \;,r_{i,m}^{new}\right\}$ for the *i*-th gene is taken as input. For the *j-*th rank, let $\beta_{j,m}\left(r_{\left(i\right)}^{new}\right)$ denote the probability of ${\hat{r}}_{j}\leq r_{i,j}^{new}$ under the null distribution and $\beta_{j,m}\left(r_{\left(i\right)}^{new}\right)=\sum_{l=j}^{m}C_{m}^{l}{\left(r_{i,j}^{new}\right)}^{l}{\left(1-r_{i,j}^{new}\right)}^{m-l}$ Since some rank lists may contain large variation from the true rank, Kolde *et al*. [2] proposed to use the minimum value of $\beta_{j,m}\left(r_{\left(i\right)}^{new}\right)$ over *j *= 1, 2,⋯,*m*, that is, $\rho \left(r_{\left(i\right)}^{new}\right)=\text{min}\left\{\beta_{j,m}\left(r_{\left(i\right)}^{new}\right),j=1,\cdots \;,m\right\}$ to rank the genes.

The values of $Q\left(r_{\left(i\right)}^{new}\right)$ and $\rho \left(r_{\left(i\right)}^{new}\right)$ are then used to aggregate the updated rank lists using Endeavour and RRA, respectively. Combining the two network incorporation procedures and two rank aggregation procedures together, we have four methods: CGI_Endeavour, CGI_RRA, GR_Endeavour, and GRI_RRA. We are interested in knowing which combinations of network rank list updating methods (CGI or GR) and rank list aggregation methods (Endeavour or RRA) yield the most biologically meaningful rank.

### Set the parameters for CGI and GR

The tuning parameters (τ,λ) for CGI and *d *for GR need to be set before implementing the methods. To do so, we need a set of genes, referred as training genes, known to be associated with a trait of interest. A good ranking method should put the training genes on the top. Therefore, for fixed parameters in CGI or GR, we use Wilcox rank sum statistic to test the hypothesis that the training genes are ranked higher. The ranks of these training genes are compared to that of the other genes and a p-value is obtained. Lower p-value corresponds to better performance of a rank aggregation method. Therefore, we choose the parameters that yield the lowest p-value.

### Data: gene expression profiles and protein interactions

Three gene expression data sets are used in this study: yeast compendium knockout (Compendium) [28], cell cycle (Cycle) [29,30] and stress response (Stress) [31]. The Compendium data consists of yeast gene expression profiles across 300 conditions. The cell cycle data includes expression profiles of yeast genes at 17 time points across five cell cycles. The stress response data contains expression profiles across 173 diverse environmental conditions such as temperature shocks, amino acid starvation and nitrogen source depletion.

To study the effects of different networks, three yeast protein interaction networks from BioGRID [32], DIP [33] and MIPS [34] are used. The BioGRID (3.2.101 version) network contains 6,226 proteins and 220,823 interactions. The DIP [33] network (20130707 version) consists of 4,767 proteins and 22,162 interactions. The MIPS [34] network (18052006 version) includes 4,554 proteins and 12,526 interactions. It can be seen that the BioGRID network contains many more genes and interactions than either DIP or MIPS. In addition, we also form an integrated network by combing all the interactions from the three networks which contains 6,567 proteins and 229,773 interactions.

### Evaluation criteria for the different rank aggregation methods

We study the effectiveness of integrating networks with rank lists using C-GLEndeavour, CGI_RRA, GR_Endeavour, and GR_RRA based on yeast gene expression and protein interactions and compare their performance with the corresponding Endeavour and RRA without integrating network information. In addition, we also study the performance of first integrating the rank lists by either Endeavour or RRA and then integrating the resulting list with the network by CGI or GR. These methods are denoted as Endeavour_CGI, Endeavour_GR, RRA_CGI, and RRA_GR, respectively. We conceive a study of genes having the same gene ontology (GO) as a set of transcription factors (TF) having the same GO term. For a GO term, we first collect all the TFs having the term and take all the genes having the same GO term as the TFs as the golden positive set. Genes with highly correlated expression profiles with the TF are more likely to have the same GO function as the TF. Therefore, we rank the genes according to their co-expression level with the TF with high absolute correlation ranked high on the list. For each TF with a given GO term, a rank list is obtained and thus, a set of rank lists is obtained for each GO term. A total of 269 TFs from [35] are used in this study.

Given the gene expression, protein-protein interaction network and known associations between genes and GO terms, we evaluate whether the network tuned rank aggregation methods (CGI_Endeavour, CGI_RRA, GR_Endeavour and GR_RRA) perform better than the original aggregation methods (Endeavour and GR). We adopt a large scale 10-fold cross-validation for the comparison. More specifically, we randomly divide the known genes of a GO term into 10 subsets with roughly equal number of genes. Therefore, each subset contains about 10% of known genes having the GO term. In each run, we assume that one subset of genes is already known and use them as training set to select the parameters for CGI and GR. The remaining 9 subsets of genes are used as validation set and ranked against the other genes based on the selected parameters. Repeating this procedure for each subset, we obtain 10 rank lists and 10 p-values by comparing the ranks of the validation set of genes with the remaining genes using Wilcox rank sum test for each GO term. We repeat this process 10 times to obtain a total of 100 rank lists and p-values for the combination of each GO term and each method. Lower p-values indicate better performance of an ranking method.

We also study the effect of incompleteness, false positive and false negative interactions in networks on the performance of the integration methods by permuting some of the interactions in the network.

## Results

We mainly present our results with the combination of the Compendium gene expression data and BioGRID interaction data in the main text. The results based on other expression and protein interaction data are presented in the Additional Files. We download the TFs for each GO term and the known associations between protein and GO terms from GO slim in the Saccharomyces Genome Database [36]. We retain the GO terms with at least 30 known genes and 5 TFs so that appropriate statistical analysis can be carried out. The GO terms with more than 40 TFs are filtered out to control the computation time. We only keep the GO terms indicating significant result (p-value < 1 × 10^-4^) with the original rank aggregation method by either Endeavour or RRA. As a result, a total of 18 GO terms are retained.

### Comparison of network tuned rank aggregation methods with the original rank aggregation methods of Endeavour and RRA

We first compare the performance of Endeavour and GR using gene expression data only. We then compare the performance of CGI_Endeavour, CGI_RRA, GR_Endeavour, and GR_RRA, together with Endeavour and RRA, using the evaluation procedures described in subsection 2.7.

**Figure 1:**
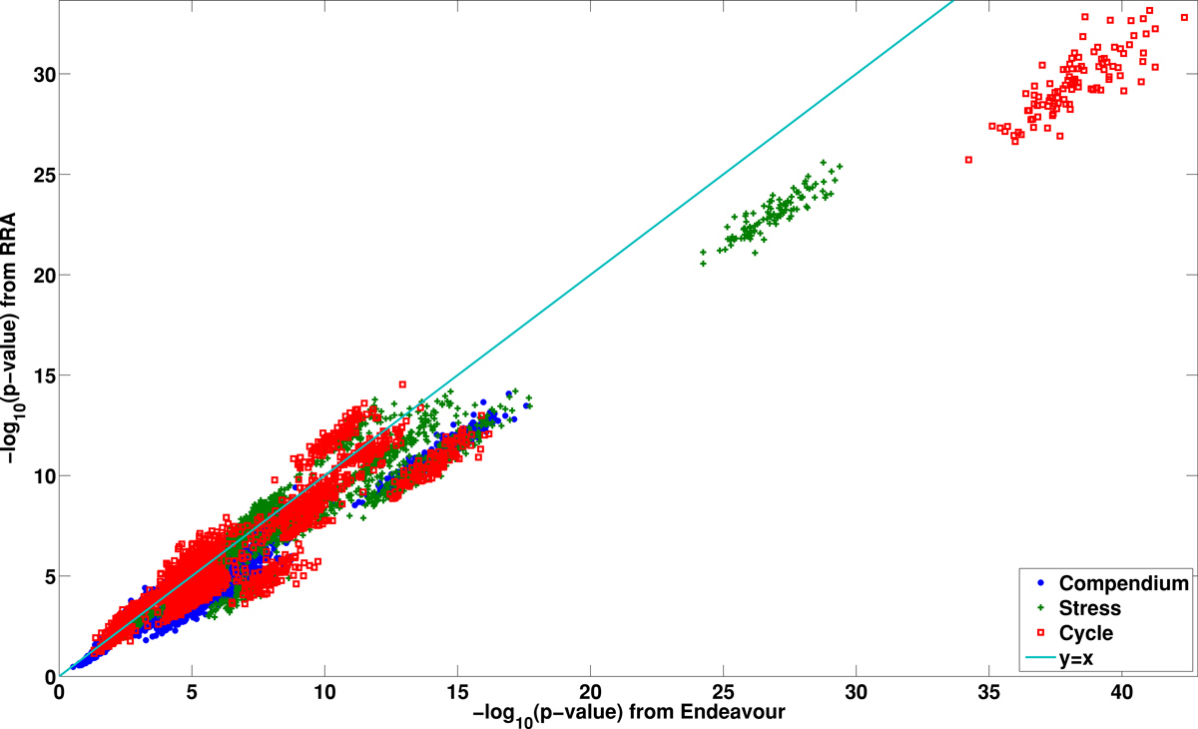
**Comparison of the performance of Endeavour and RRA based on the Compendium, Stress and Cell Cycle expression data sets**. The x-axis indicates - log_10_(p-value) from Endeavour and the y-axis is - log_10_(p-value) from RRA. The - log_10_(p-value) from Endeavour is generally larger than that from RRA indicating slightly better performance of Endeavour over RRA based on these data sets.

Figure 1 shows the relative performance of Endeavour and RRA based on the three different expression data sets. Each point corresponds to - log_10_(*p *- *value*) of a GO term from one of 10 rounds of 10-fold cross validation experiments. It can be seen that Endeavour outperforms RRA in most situations across all three expression data sets as the value of - log_10_(*p *- *value*) from Endeavour is generally larger than that from RRA.

**Figure 2:**
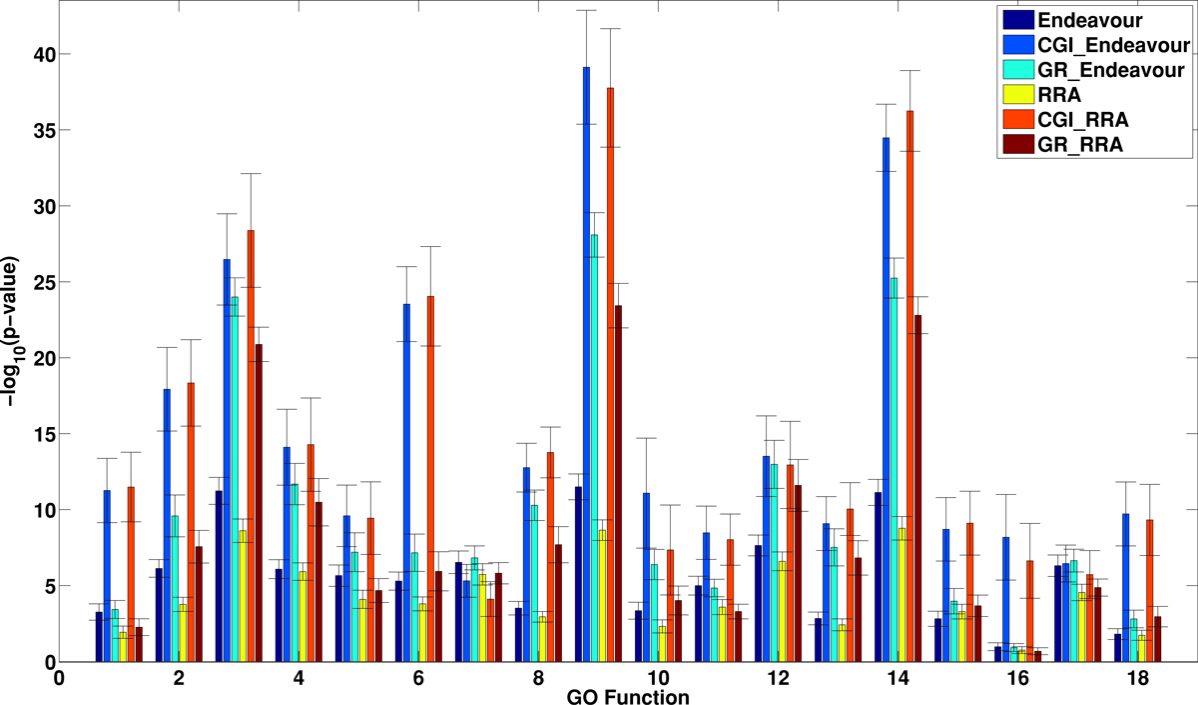
**The average - log_10_(*p *- *value*) together with standard errors of six rank aggregation methods: Endeavour, CGI_Endeavour, GR_Endeavour, RRA, CGI_RRA, and GR_RRA based on the Compendium expression data and BioGRID interactions**. The x-axis shows the selected 18 GO function terms and the y-axis indicates - log_10_(*p *- *value*) of the different methods. Higher value corresponds to better performance.

Figure 2 shows the average - log_10_(p - *value*) together with the standard errors of the four integrative approaches, CGI_Endeavour, CGI_RRA, GR_Endeavour and GR_RRA, by incorporating network information with CGI and GR based on the combination of the compendium expression data and the BioGRID protein interactions. For comparison, the results for Endeavour and RRA are also shown. We have the following observations. First, except for GO term 7 (invasive growth in response to glucose limitation), the average - log(*p *- *value*) based on both C-GLEndeavour and GR_Endeavour are higher than that for Endeavour, indicating the usefulness of incorporating network information for rank aggregation. Second, the improvement of CGI_Endeavour over Endeavour is much higher than that for GR_Endeavour indicating that CGI makes more effective use of network than GR integrating rank lists with the network. Third, the relative performance of RRA, CGI_RRA, and GR_RRA are similar to that among Endeavour, CGI_Endeavour, and GR_Endeavour. Fourth, the performances of CGI_Endeavour and CGI_RRA are similar across the 18 GO categories under study.

Figures S1-S8 in the Additional File show the corresponding results with other combinations of gene expression data and protein interaction networks. The relative performance of the six rank aggregation methods are the same as presented above. However, it is noted that the average - log(*p *- *value*) based on the DIP and MIPS protein interaction data sets are generally smaller than that based on the BioGRID network. The results are most likely due to the relative sparse yeast networks in DIP and MIPS compared to BioGRID. Thus, for the purpose of rank aggregation, we recommend the use of the BioGRID interaction data set.

We will mainly focus on CGI_Endeavour and CGI_RRA in the rest of this paper. The average selected parameters for CGI_Endeavour and CGI_RRA are presented in Tables 1 and 2 in Additional File. We will use the same parameters in the following analysis.

**Table 1:** The improvement by incorporating network using CGI_Endeavour and CGI_RRA is significantly correlated with the number of training genes.

		CGI_Endeavour		CGI_RRA	
**Expression**	**Network**	**correlation**	**p-value**	**correlation**	**p-value**

Compendium	BioGRID DIP MIPS	0.6614(0.0943) 0.722(0.0796) 0.4212(0.1339)	0.0041(0.0065) 0.0015(0.0040) 0.0668(0.0830)	0.6581(0.0920) 0.7485(0.0855) 0.5232(0.0995)	0.0041(0.0072) 0.0013(0.0046) 0.0217(0.0271)
Stress	BioGRID DIP MIPS	0.6313(0.0865) 0.6636(0.13300) 0.4620(0.1000)	0.0060(0.0119) 0.0084(0.0212) 0.0387(0.0397)	0.6387(0.0874) 0.7060(0.1333) 0.3961(0.1160)	0.0055(0.0114) 0.0062(0.0162) 0.0725(0.0739)
Cycle	BioGRID DIP MIPS	0.7776(0.0894) 0.6497(0.1376) 0.5997(0.0823)	0.0007(0.0019) 0.0104(0.0248) 0.0082(0.0131)	0.6585(0.1071) 0.5645(0.1482) 0.5414(0.0890)	0.0063(0.0151) 0.0227(0.0342) 0.0170(0.0233)

The table shows the average Spearman correlation between log-p-fold and the number of training genes together with the standard deviation (3rd and 5th columns) and the mean p-value and its standard deviation (4th and 6th columns) using all combinations of gene expression and interaction data sets.

Elements in the parenthesis are standard deviation.

**Table 2:** Computational time of implementing CGI_Endeavour, CGI_RRA, GR_Endeavour and GR_RRA with the Compendium expression data and the BioGRID interaction network for the GO term of ATPase activity.

Method	Time
CGI_Endeavour	1:11:31

CGI_RRA	43:14

GR_Endeavour	6:54:33

GR_RRA	6:53:07

### The effect of the number of the training genes on the performance of rank aggregation methods

In our studies, we determine the parameters using training genes so that they are most likely to be ranked on the top. It is expected that the improvement of our methods using networks over the ones without networks increases with the number of training genes. We use the log-ratio of the p-value from the method without using the network over the p-value from the method integrating the network to evaluate the improvement of our integrative method and denote this quantity as "log-p-fold". The higher this value is, the more effective of using the network information becomes. Positive value of log-p-fold indicates improvement of integrating networks. Therefore, for a given method of combining express data and network, we calculate the Spearman correlation between the log-p-fold values and the number of training genes across the 18 GO terms for each of the 10 rounds of 10-fold cross validation experiments. The mean and standard deviation of these Spearman correlations are calculated. The results for all combinations of integration method, expression data and network are given in Table 1. All the correlations are positive indicating that the improvement of using networks increases with the number of training genes for CGI_Endeavour and CGI_RRA. All mean p-values calculated based on the alternative hypothesis that the correlation is positive indicates that the correlations are all significantly higher than zero.

From the above analysis, we see that the standard deviations calculated across 10 rounds of the 10-fold cross validation experiments are small. Considering the huge time consuming burden for implementing 10 rounds of 10-fold experiments, we present the following results with only one fixed classification of training gene set and validation gene set.

### The contribution of network to the network tuned rank aggregation methods

**Figure 3:**
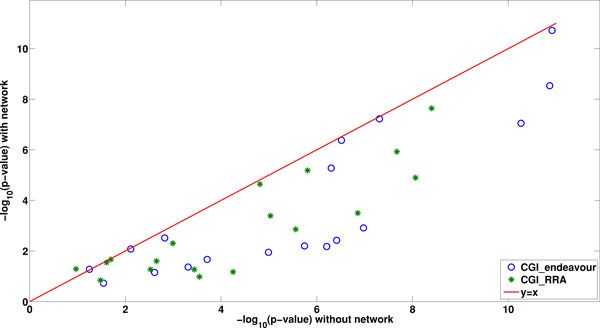
**The effects of randomized gene labels on the performance of CGI_Endeavour and CGI_RRA based on the Compendium expression and the BioGRID network**. The x-axis is - log_10_(*p *- *value*) from the original method. The y-axis is - log_10_(*p *- *value*) from the methods incorporating network information.

We study if the observed improvement of rank aggregation methods incorporating networks was due to the added biological information in the protein interaction network. Following the procedures implemented in [20], we randomly permute gene names in the network, and use the rank lists together with the permuted network for incorporating network information and aggregating the updated rank lists. The randomized networks preserve the topology of the original network, but dissociate any correlation between the network and the rank lists. Figure 3 shows the relationship between the - log_10_(*p *- *value*) using the randomized network based on BioGRID and the Compendium expression data using CGI_Endeavour and CGI_RRA and that without using the network. It can be seen that the - log_10_(*p *- *value*) using the randomized network is smaller than that without the network, indicating that the randomized network can decrease the performance if the network is random as expected. The results also show that the observed improved performance using true networks is due to the contributions of the molecular network. We also carry out the same analysis using other gene expression and protein interaction data sets and the results are given in Additional File (Figures S9-S16).

### The effects of false interactions in PPI on the performance of network tuned rank aggregation methods

**Figure 4:**
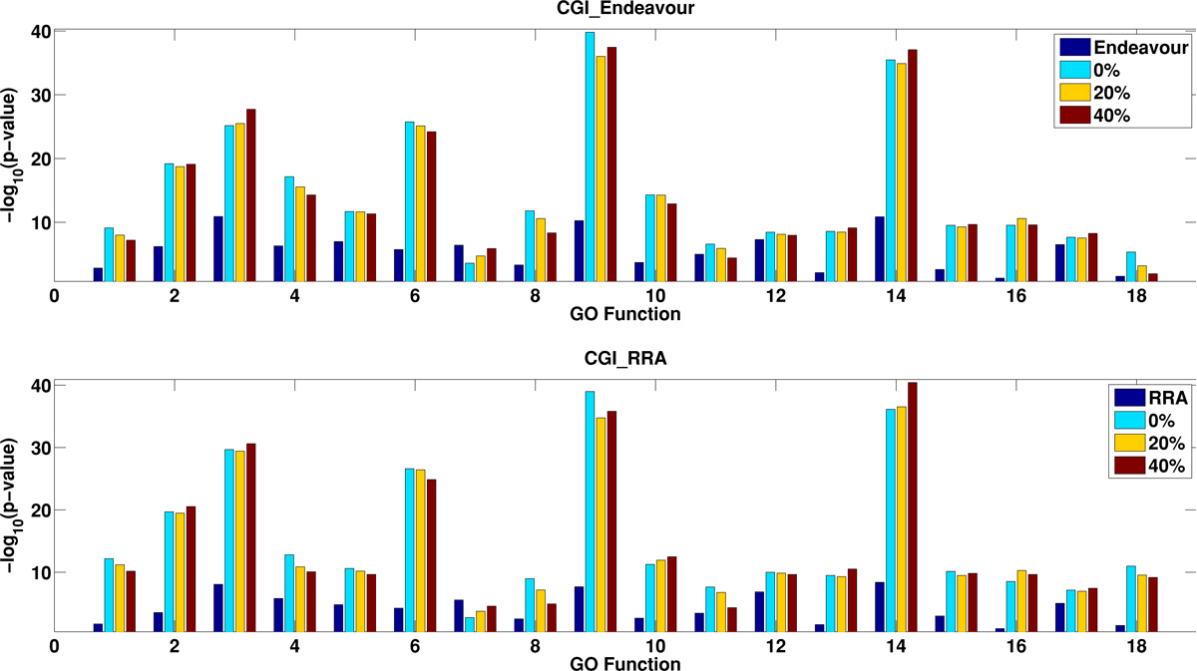
**The effect of noise in PPI network on the performance of CG_Eendeavour and CGI_RRA based on the Compendium expression and the BioGRID network**. The x-axis shows the selected 18 GO function terms and the y-axis indicates - log_10_(*p *- *value*) from the different methods. Higher value corresponds to better performance.

It is well known that most current available PPI networks are incomplete and consist of many false interactions. We investigate how false interactions in PPI networks affect the performance of network tuned rank aggregation methods. Therefore, we study CGI_Endeavour and CGI_RRA based on networks with different levels of false interactions. As in [1], we randomly select *α% *of the edges and replaced them with randomly selected protein pairs with no interactions in the original data set. Then we implement CGI_Endeavour and CGI_RRA based on the new network and input rank lists. We perform this procedure for noisy level of 20% and 40% of the total edges for each network. Figure 4 shows the log-p-fold for different GO terms based on CGI_Endeavour and CGI_RRA using the noisy network generated from BioGRID and the compendium expression data. For most of GO function terms, the performance of CGI_Endeavour and CGI_RRA decreases as the noise level increases as expected. Even at 40% noise level, their performance is still better than not using the networks. The results based on other combinations of noisy networks and expression data sets are presented in the Additional File as Figures S17-S24.

### Performance of CGI_Endeavour, GFLEndeavour, CGI_RRA and GR_RRA using the integrated network

**Figure 5:**
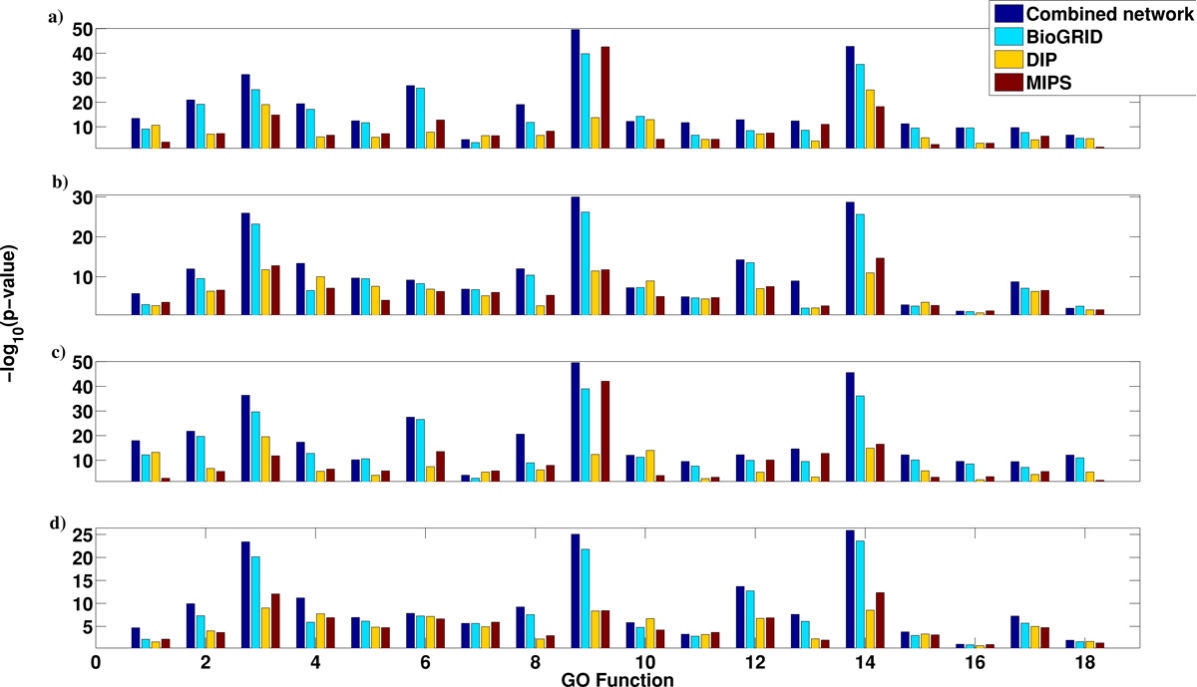
**Comparison of the performance of the integration methods based on the integrated and the individual networks**. The four sub-figures (from top to bottom) indicate the performance of CGI_Endeavour, GR_Endeavour, CGI_RRA & GR_RRA based on the integrated, BioGRID, DIP and MIPS interaction networks and the Compendium gene expression data.

The performances of our integration methods based on the BioGRID network are better than that based on the DIP and the MIPS networks. We wonder if the integrated network by combining all the interactions from these three networks can further improve the performance of our integration approaches. Therefore, we merge the three networks together and run our methods based on the integrated network. Figure 5 shows the performance of a) CGI_Endeavour, b) GR_Endeavour, c) CGI_RRA and d) GR_RRA based on the integrated, BioGRID, DIP and MIPS networks and the Compendium expression data. We can see that the performances of the integration method based on the integrated network are better than that using any individual network.

### An alternative strategy of firstly aggregating rank lists and then integrating network

**Figure 6:**
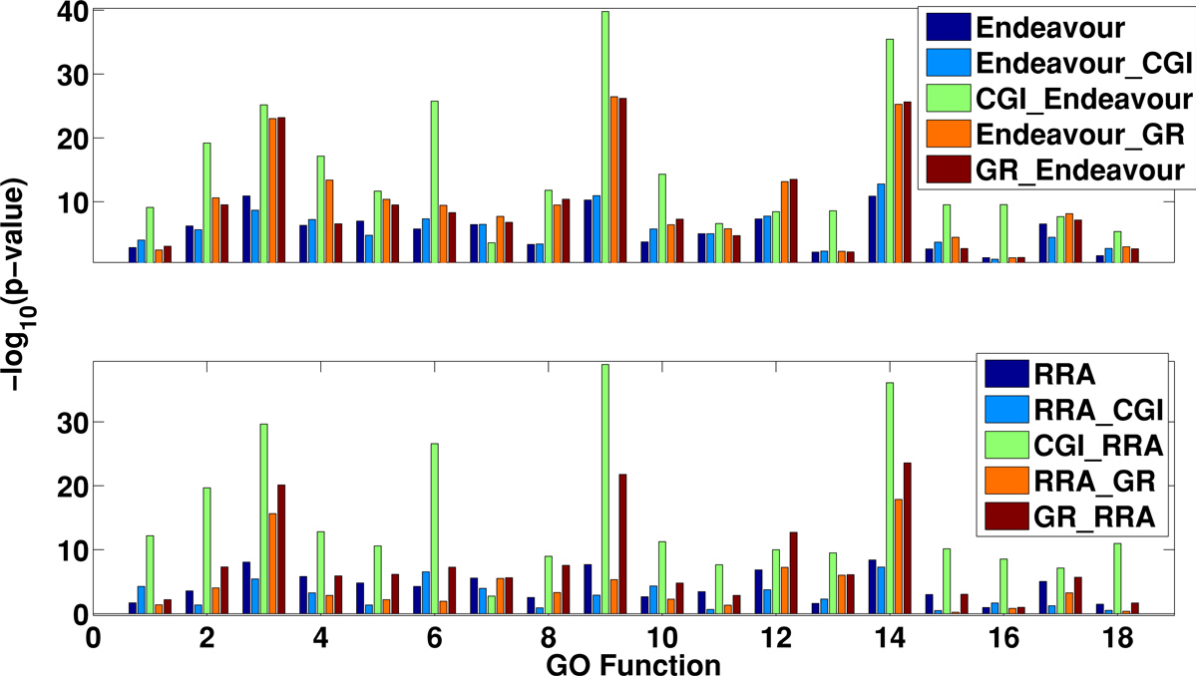
**Comparison of the performance of CGI_Endeavour, GR_Endeavour, CGI_RRA and GR_RRA with the corresponding methods first integrating the rank lists followed with network update**. The BioGRID network and the Compendium gene expression data are used.

In the above studies, we first integrate each individual rank list with the network and then aggregate the updated rank lists. A natural alternative approach is to first aggregate rank lists and then update the integrated rank list with the network. Corresponding to our four methods, we name the four methods following the alternative approach as Endeavour_CGI, Endeavour_GR, RRA_CGI and RRA_GR. Based on the BioGRID network and the Compendium expression data, we compare the performance of these four methods with the approaches studied above. Figure 6 shows the relative performances of the Endeavour based methods (upper panel) and the RRA based methods (lower panel), respectively. It shows that CGI_Endeavour and CGI_RRA are the best performers in each class, respectively. While CGI_Endeavour and CGI_RRA perform similarly across all the 18 GO categories.

### Computational time

The computational time depends on the number of individual rank lists to be integrated. We compare the computational time of CGI_Endeavour, GR_Endeavour, CGI_RRA and GR_RRA for a specific GO function: ATPase activity, based on the BioGRID network and the Compendium expression data. There are 5 known transcription factors for this function indicating 5 individual rank lists to be aggregated. Table 2 shows the time spent by these four methods using a computer of CPU speed of 2.3 GHz and 16 Gb memory. CGI_Endeavour and CGI_RRA are more time efficient than GR_Endeavour and GR_RRA. RRA related methods are more time efficient than Endeavour related methods, consistent with the conclusion in [2]. We do not include the diffusion kernel matrix computation time in the time of CGI related method. Because once we have got the diffusion kernel matrix from the network, we can use it repeatedly for network integration of different rank lists. Whereas GeneRank need to be rerun each time for each network integration. Therefore, CGI related methods are more time efficient.

### Case studies: identification of pathway genes from knock-out experiments

Kolde *et al*. [2] validated their RRA method by predicting the pathway members with knock-out data. Genes with large absolute differential expression levels before and after a TF is knocked out are ranked high. Rank lists of genes whose expression level were most affected by knockout of a TF are directly extracted from [37].

**Figure 7:**
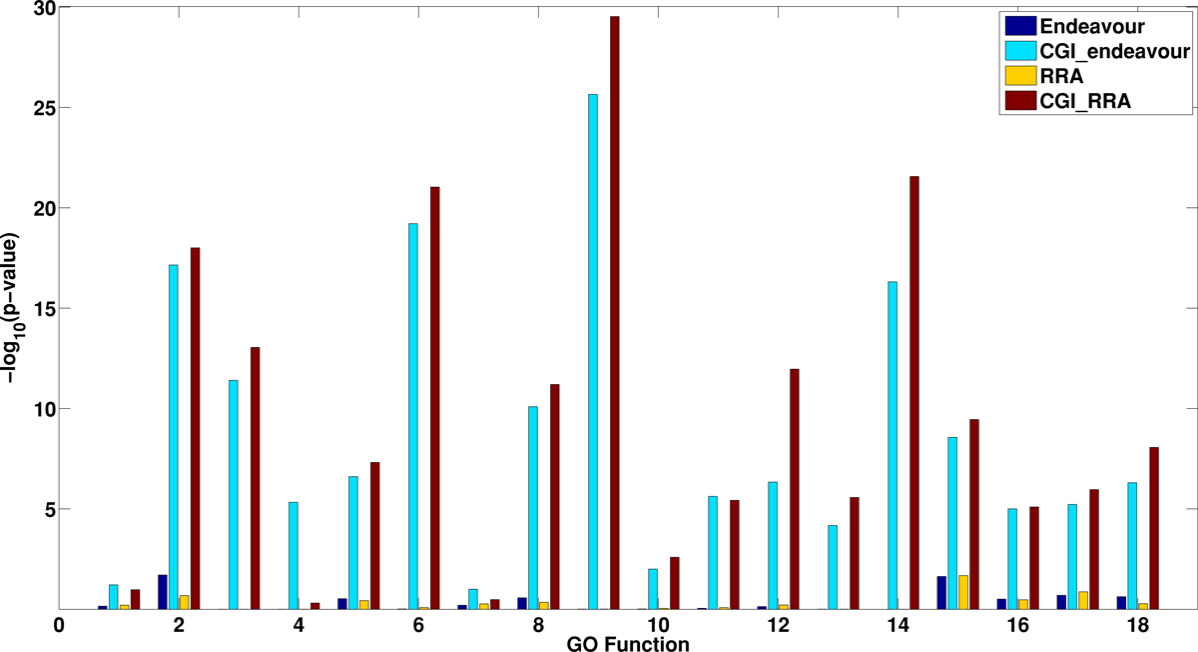
**Comparison of the performance of Endeavour, CGI_Endeavour, RRA and CGI_RRA on predicting pathway members based on TF knockout data from **[37]**and the BioGRID interactions**. The x-axis shows the selected 18 GO function terms and the y-axis indicates - log_10_(*p *- *value*) from the different methods. Higher value corresponds to better performance.

For a specific GO function term (interchangeable with pathway in their paper), Kolde et al. [2] identified the known TFs and collected the corresponding rank lists. They showed that the integrated rank list resulted from RRA is better than any single rank list corresponding to a TF. We validate our method by comparing the results from direct aggregation using RRA or Endeavour and aggregation after incorporating the BioGRD network information using 10% of the genes having the same GO term as training set. We select the same 18 GO terms as above. Figure 7 shows - log_10_(*p *- *value) *for the Endeavour, CGI_Endeavour, RRA, and CGI_RRA based on BioGRID. It can be seen that CGI_Endeavour and CGI_RRA perform much better than Endeavour and RRA, respectively. The results using MIPS and DIP interaction networks are given in Additional File (Figures S25-S26).

## Discussion and conclusion

Gene prioritization continues to play important roles in the identification of genes responsible for complex traits, finding targets of gene regulators such as TFs, microRNAs or long non-coding RNAs, and construction of gene regulation networks. Many methods have been developed to integrate multiple rank lists from similar types of studies such as gene expression profiles or one gene expression study with a network. However, integrating multiple rank lists with one or more networks for gene prioritization is understudied. Here we propose to first update each rank list with a network using similar ideas as in CGI or GR and then integrate the updated gene rank lists using Endeavour or RRA. Using three yeast gene expression data sets and three protein interaction networks, we study the effectiveness of the approaches for integrating multiple lists with networks. We show that integrating multiple rank lists with a PPI network can indeed improve the performance of rank list aggregation over corresponding methods without using network information. As expected, the extent of improvement depends on the network used. It is shown that among the three yeast interaction networks studied in this paper, the BioGRID network achieves the largest improvement compared to DIP and MIPS, most likely due to the large size of the BioGRID network. It is also shown that the combination of CGI with Endeavour or RRA generally outperforms GR combined with Endeavour or RRA. On the other hand, the performance of CGI_Endeavour is similar to CGI_RRA. The conclusions hold for all the three gene expression data sets studied indicating the generality of these results.

We then show that the improvement of CGI_Endeavour or CGI_RRA incorporating network information over Endeavour or RRA, respectively, is indeed due to the incorporation of network. If the gene labels are randomized while keeping the network structure unchanged, it is shown that the performance of CGI_Endeavour or CGI_RRA would be worse than Endeavour or RRA, respectively. Finally, we show that the performance of CGI_Endeavour or CGI_RRA is relatively stable with many false positive and false negative interactions.

Our methods are validated based on rank lists obtained according to the gene co-expression level with the TF for a given GO term. Actually, these methods are applicable to rank lists acquired from other methods.

Our study has several limitations. First, we use yeast gene expression and protein interaction data for our study because the interaction data is relatively rich compared to other organisms such as human. It is not clear whether the conclusions derived from this study are valid to many other organisms such as human where our knowledge of the interaction network is much less than for yeast. On the other hand, more and more interaction data will be generated for different organisms and we expect that our results will be applicable as more interaction data become available. Second, the PPI networks including BioGRID, DIP and MIPS we study in this paper are static and do not indicate where and when the interactions occur. In reality, protein interactions are dynamic depending on particular cellular locations, tissues, and environment. For certain protein properties, if the conditions or tissues for the properties to show up are known, we may restrict to such networks under these conditions instead of the whole static network. Third, we determine the parameters in CGI or GR based on a set of known genes associated with a trait of interest. It is not clear how to best set the parameters when training genes are not available. Finally, we only consider one network in this study. Multiple networks are usually available and it is not clear whether one should first derive one comprehensive network and then use the approaches developed here or alternatively, design new approaches to simultaneously integrate multiple networks. These are problems for further studies.

In conclusion, integrating multiple rank lists together with network information using CGLEndeavor or CGI_RRA can be effectively used for gene prioritization. The extent of improvement over corresponding approaches without using network information depends on the completeness and accuracy of the network. Even for relative sparse networks containing a significant fraction of false positives, CGI_Endeavour and CGI_RRA still outperform their corresponding counterparts, Endeavour and RRA, respectively.

## Competing interests

The authors declare that they have no competing interests.

## Authors' contributions

FS conceived and designed the study. WW carried out the experiments, analyzed the results, and drafted the paper. XZ and ZL helped with the experimental design and interpretation of the results. All authors participated in finalizing the paper, read the manuscript and approved the final version.

## Supplementary Material

Additional file 1 **Supplementary**.pdf includes all the supplementary figures and tables. Click here for file

## Declarations

The cost of publication of this paper comes from US National Institutes of Health Center for Excellence in Genomic Sciences [P50HG002790].

This article has been published as part of *BMC Bioinformatics *Volume 16 Supplement 1, 2015: Selected articles from the Thirteenth Asia Pacific Bioinformatics Conference (APBC 2015): Bioinformatics. The full contents of the supplement are available online at http://www.biomedcentral.com/bmcbioinformatics/supplements/16/S1
