# Supplementary material for: Network tuned multiple rank aggregation and applications to gene ranking
Source: BMC Bioinformatics. 2015 Jan 21;16(Suppl 1):S6. doi: 10.1186/1471-2105-16-S1-S6 (PMC4331705; doi:10.1186/1471-2105-16-S1-S6)
Supplement: Additional file 1 — Supplementary.pdf includes all the supplementary figures and tables. [file 1471-2105-16-S1-S6-S1.pdf]

Network Tuned Multiple Rank Aggregation and Applications to Gene Ranking

Wenhui Wang, Xianghong Jasmine Zhou, Zhenqiu Liu and Fengzhu Sun *

November 20, 2014

Figures S1-S8 show the results of Endeavour, RRA, CGI_Endeavour, CGI_RRA, GR_Endeavour and GR_RRA based on different combinations of gene expression data sets and protein interaction networks.

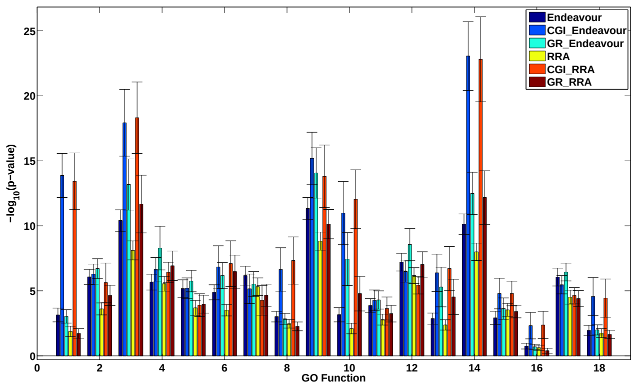

Figure S1: **The average $-\log_{10}(p - value)$ together with standard errors of six rank aggregation methods: Endeavour, CGI_Endeavour, GR_Endeavour, RRA, CGI_RRA, and GR_RRA based on the Compendium expression data and DIP interactions.** The x-axis shows the selected 18 GO function terms and the y-axis indicates $-\log_{10}(p - value)$ from the different methods. Higher value corresponds to better performance.

*To whom correspondence should be addressed

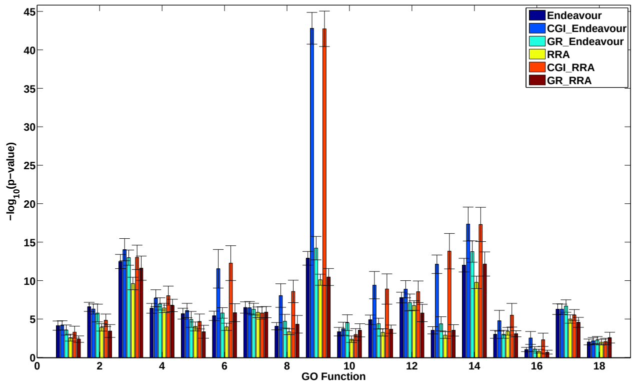

Figure S2: **The average $-\log_{10}(p - value)$ together with standard errors of six rank aggregation methods: Endeavour, CGI_Endeavour, GR_Endeavour, RRA, CGI_RRA, and GR_RRA based on the Compendium expression data and MIPS interactions.** The x-axis shows the selected 18 GO function terms and the y-axis indicates $-\log_{10}(p - value)$ from the different methods. Higher value corresponds to better performance.

Figures S9-S16 show the effects of randomized gene labels on the performance of CGI_Endeavour and CGI_RRA based on the different gene expression and protein interaction data.

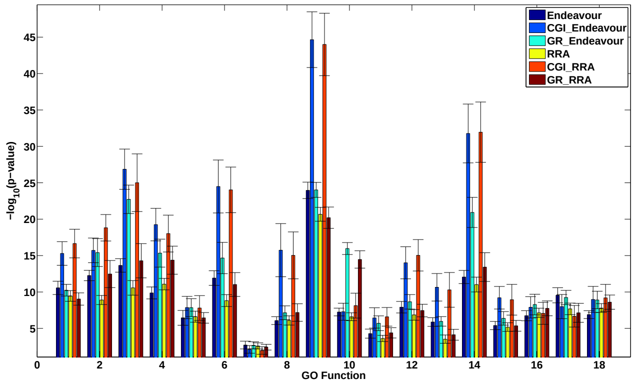

Figure S3: **The average $-\log_{10}(p - value)$ together with standard errors of six rank aggregation methods: Endeavour, CGI_Endeavour, GR_Endeavour, RRA, CGI_RRA, and GR_RRA based on the Stress expression data and BioGRID interactions.** The x-axis shows the selected 18 GO function terms and the y-axis indicates $-\log_{10}(p - value)$ from the different methods. Higher value corresponds to better performance.

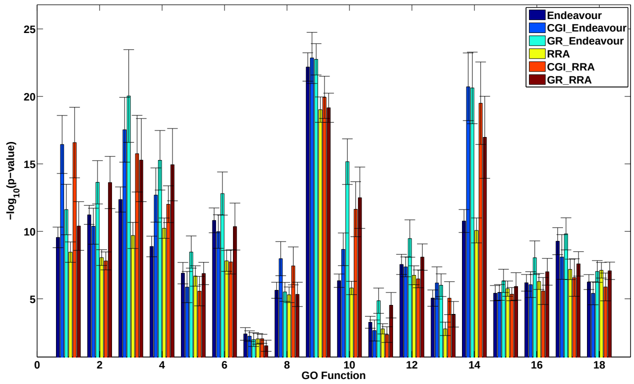

Figure S4: **The average $-\log_{10}(p - value)$ together with standard errors of six rank aggregation methods: Endeavour, CGI_Endeavour, GR_Endeavour, RRA, CGI_RRA, and GR_RRA based on the Stress expression data and DIP interactions.** The x-axis shows the selected 18 GO function terms and the y-axis indicates $-\log_{10}(p - value)$ from the different methods. Higher value corresponds to better performance.

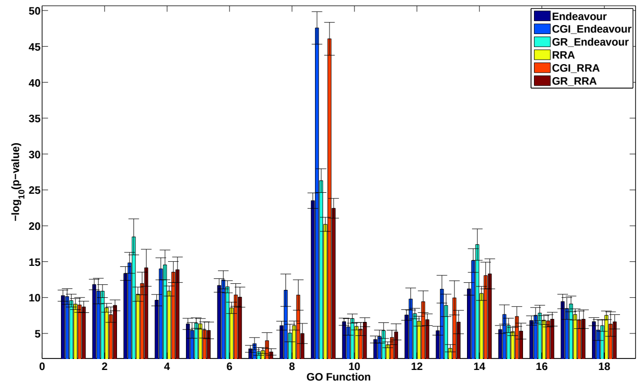

Figure S5: **The average $-\log_{10}(p - value)$ together with standard errors of six rank aggregation methods: Endeavour, CGI_Endeavour, GR_Endeavour, RRA, CGI_RRA, and GR_RRA based on the Stress expression data and MIPS interactions.** The x-axis shows the selected 18 GO function terms and the y-axis indicates $-\log_{10}(p - value)$ from the different methods. Higher value corresponds to better performance.

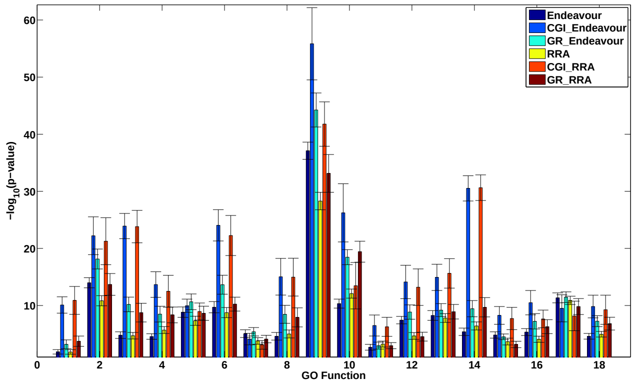

Figure S6: **The average $-\log_{10}(p - value)$ together with standard errors of six rank aggregation methods: Endeavour, CGI_Endeavour, GR_Endeavour, RRA, CGI_RRA, and GR_RRA based on the Cell Cycle expression data and BioGRID interactions.** The x-axis shows the selected 18 GO function terms and the y-axis indicates $-\log_{10}(p - value)$ from the different methods. Higher value corresponds to better performance.

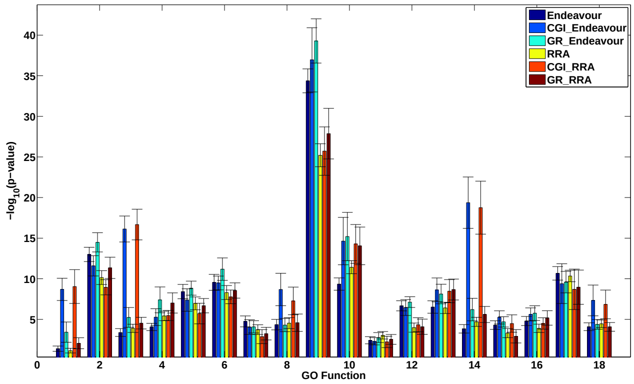

Figure S7: **The average $-\log_{10}(p - value)$ together with standard errors of six rank aggregation methods: Endeavour, CGI_Endeavour, GR_Endeavour, RRA, CGI_RRA, and GR_RRA based on the Cell Cycle expression data and DIP interactions.** The x-axis shows the selected 18 GO function terms and the y-axis indicates $-\log_{10}(p - value)$ from the different methods. Higher value corresponds to better performance.

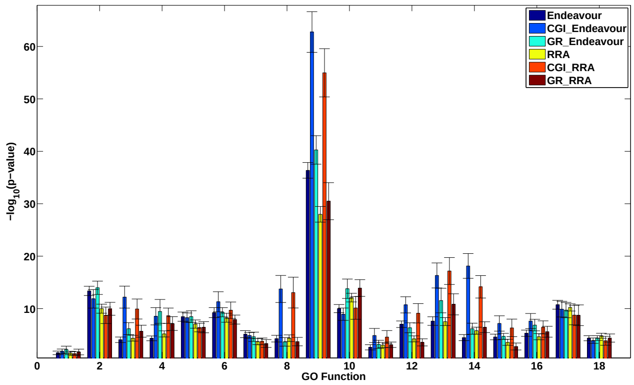

Figure S8: **The average $-\log_{10}(p - value)$ together with standard errors of six rank aggregation methods: Endeavour, CGI_Endeavour, GR_Endeavour, RRA, CGI_RRA, and GR_RRA based on the Cell Cycle expression data and MIPS interactions.** The x-axis shows the selected 18 GO function terms and the y-axis indicates $-\log_{10}(p - value)$ from the different methods. Higher value corresponds to better performance.

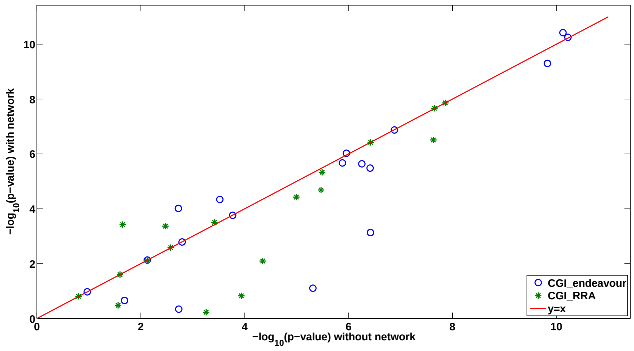

Figure S9: **The effects of randomized gene labels on the performance of CGI_Endeavour and CGI_RRA based on the Compendium expression and the DIP network.** The x-axis is $-\log_{10}(p - value)$ from the original method. The y-axis is $-\log_{10}(p - value)$ from the methods incorporating network information.

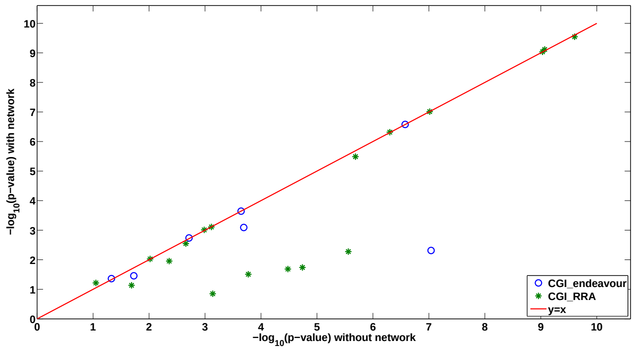

Figure S10: **The effects of randomized gene labels on the performance of CGI_Endeavour and CGI_RRA based on the Compendium expression and the MIPS network.** The x-axis is $-\log_{10}(p-value)$ from the original method. The y-axis is $-\log_{10}(p-value)$ from the methods incorporating network information.

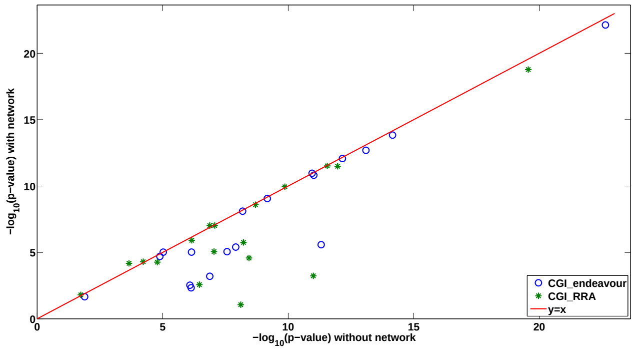

Figure S11: **The effects of randomized gene labels on the performance of CGI_Endeavour and CGI_RRA based on the Stress expression and the BioGRID network.** The x-axis is $-\log_{10}(p - value)$ from the original method. The y-axis is $-\log_{10}(p-value)$ from the methods incorporating network information.

Figures S17-S24 the effect of noise in PPI network on the performance of CGI_endeavour and CGI_RRA based on different gene expression and protein interaction data.

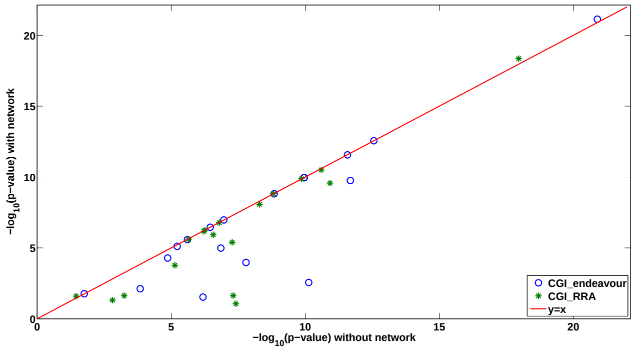

Figure S12: **The effects of randomized gene labels on the performance of CGI_Endeavour and CGI_RRA based on the Stress expression and the DIP network.** The x-axis is $-\log_{10}(p - value)$ from the original method. The y-axis is $-\log_{10}(p - value)$ from the methods incorporating network information.

Figures S25-S26 show the performance of Endeavour, CGI_Endeavour, RRA and CGI_RRA on predicting pathway members based on TF knockout data from [Reimand *et al.*, 2010] and the DIP and MIPS interactions.

References

[Reimand *et al.*, 2010] Reimand, J. *et al.* (2010) Comprehensive reanalysis of transcription factor knockout expression data in Saccharomyces cerevisiae reveals many new targets. *Nucleic Acids Res*, **38**, 4768-4777.

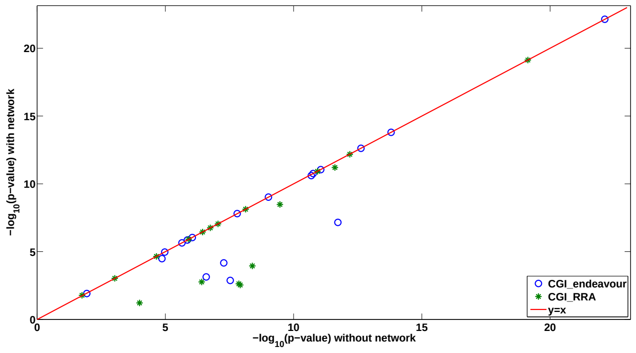

Figure S13: **The effects of randomized gene labels on the performance of CGI_Endeavour and CGI_RRA based on the Stress expression and the MIPS network.** The x-axis is $-\log_{10}(p-value)$ from the original method. The y-axis is $-\log_{10}(p-value)$ from the methods incorporating network information.

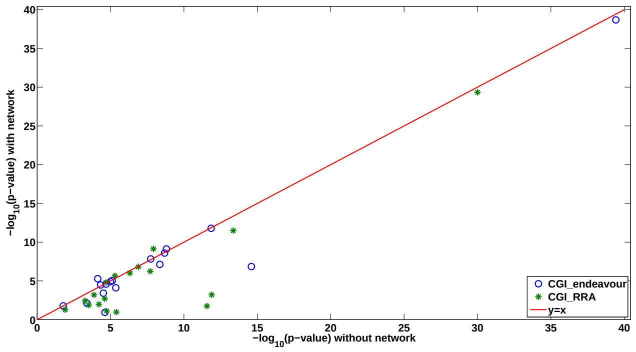

Figure S14: **The effects of randomized gene labels on the performance of CGI_Endeavour and CGI_RRA based on the Cell Cycle expression and the BioGRID network.** The x-axis is $-\log_{10}(p-value)$ from the original method. The y-axis is $-\log_{10}(p-value)$ from the methods incorporating network information.

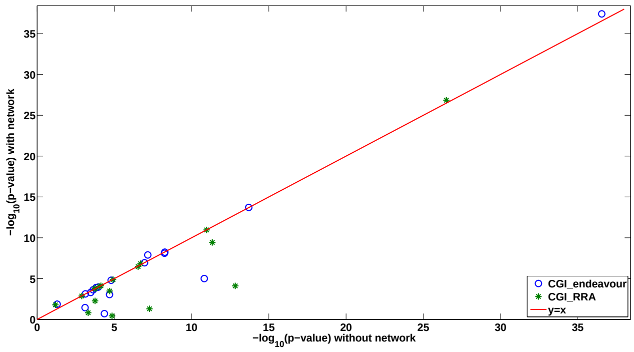

Figure S15: **The effects of randomized gene labels on the performance of CGI_Endeavour and CGI_RRA based on the Cell Cycle expression and the DIP network.** The x-axis is $-\log_{10}(p-value)$ from the original method. The y-axis is $-\log_{10}(p-value)$ from the methods incorporating network information.

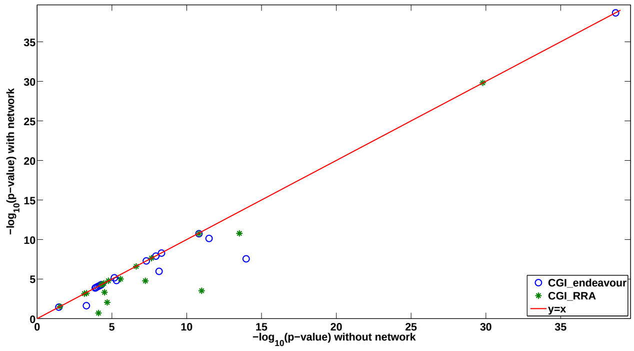

Figure S16: **The effects of randomized gene labels on the performance of CGI_Endeavour and CGI_RRA based on the Cell Cycle expression and the MIPS network.** The x-axis is $-\log_{10}(p-value)$ from the original method. The y-axis is $-\log_{10}(p-value)$ from the methods incorporating network information.

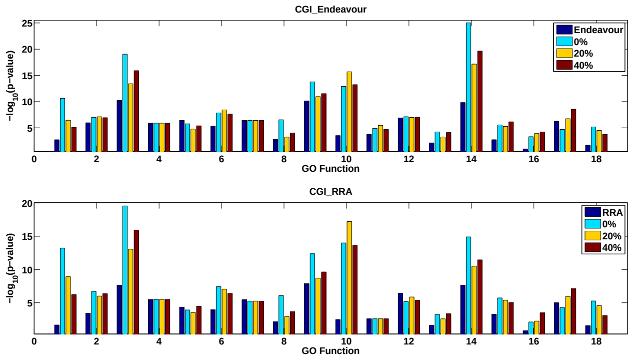

Figure S17: **The effect of noise in PPI network on the performance of CGI_endeavour and CGI_RRA based on the Compendium expression and the DIP network.** The x-axis shows the selected 18 GO function terms and the y-axis indicates $-\log_{10}(p - value)$ from the different methods. Higher value corresponds to better performance.

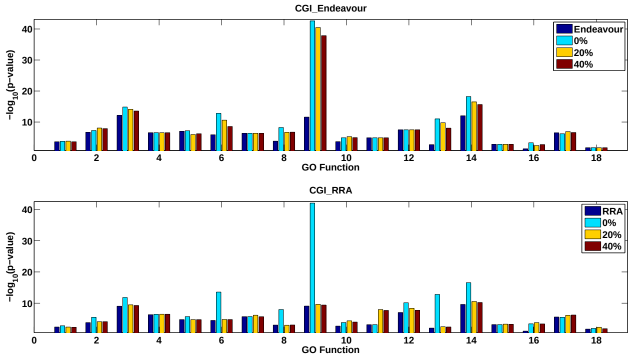

Figure S18: **The effect of noise in PPI network on the performance of CGI_endeavour and CGI_RRA based on the Compendium expression and the MIPS network.** The x-axis shows the selected 18 GO function terms and the y-axis indicates $-\log_{10}(p - value)$ from the different methods. Higher value corresponds to better performance.

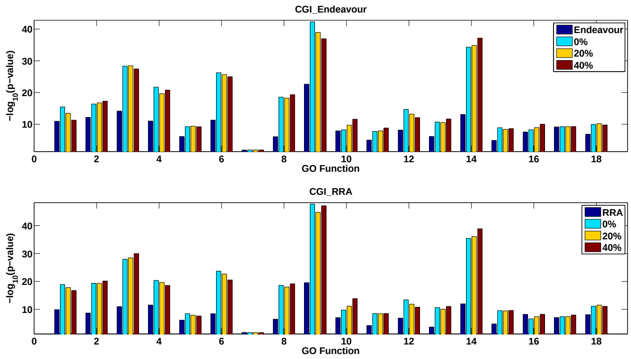

Figure S19: **The effect of noise in PPI network on the performance of CGI_endeavour and CGI_RRA based on the Stress expression and the BioGRID network.** The x-axis shows the selected 18 GO function terms and the y-axis indicates $-\log_{10}(p - value)$ from the different methods. Higher value corresponds to better performance.

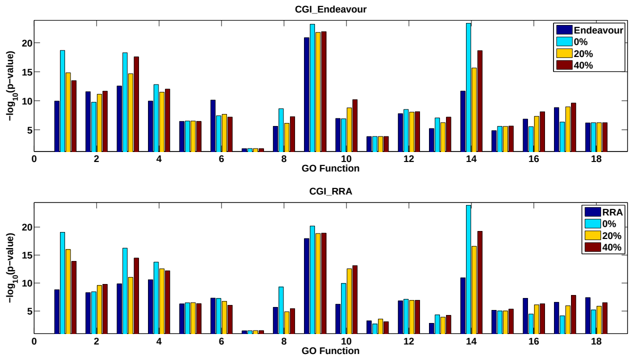

Figure S20: **The effect of noise in PPI network on the performance of CGI_endeavour and CGI_RRA based on the Stress expression and the DIP network.** The x-axis shows the selected 18 GO function terms and the y-axis indicates $-\log_{10}(p-value)$ from the different methods. Higher value corresponds to better performance.

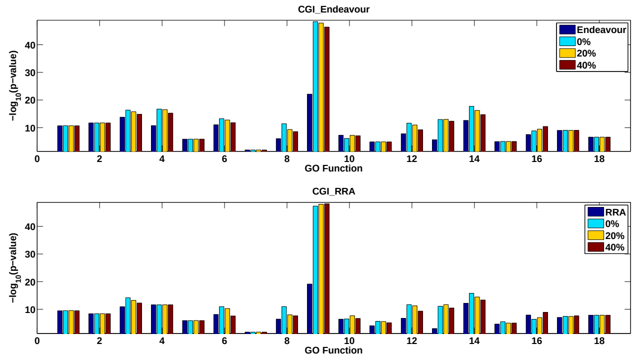

Figure S21: **The effect of noise in PPI network on the performance of CGI_endeavour and CGI_RRA based on the Stress expression and the MIPS network.** The x-axis shows the selected 18 GO function terms and the y-axis indicates $-\log_{10}(p-value)$ from the different methods. Higher value corresponds to better performance.

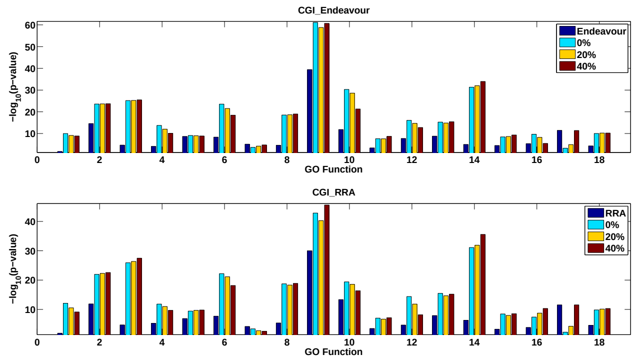

Figure S22: **The effect of noise in PPI network on the performance of CGI_endeavour and CGI_RRA based on the Cell Cycle expression and the BioGRID network.** The x-axis shows the selected 18 GO function terms and the y-axis indicates $-\log_{10}(p-value)$ from the different methods. Higher value corresponds to better performance.

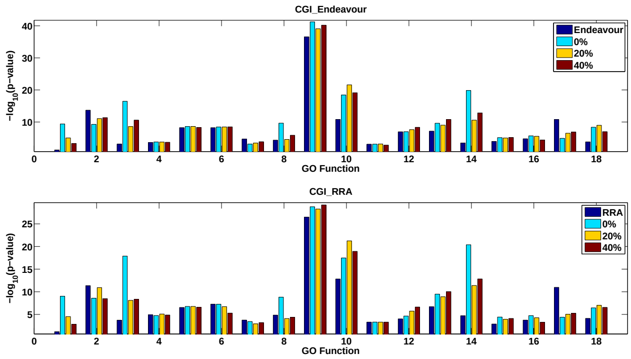

Figure S23: **The effect of noise in PPI network on the performance of CGI_endeavour and CGI_RRA based on the Cell Cycle expression and the DIP network.** The x-axis shows the selected 18 GO function terms and the y-axis indicates $-\log_{10}(p-value)$ from the different methods. Higher value corresponds to better performance.

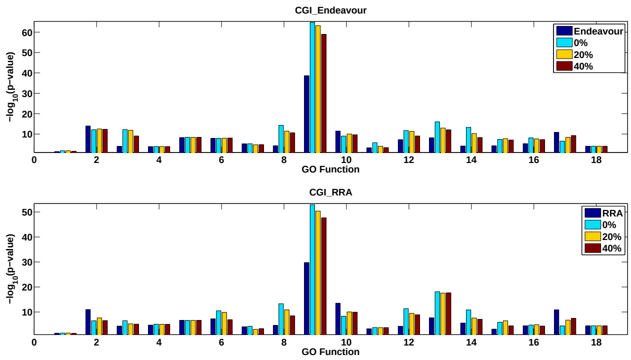

Figure S24: **The effect of noise in PPI network on the performance of CGI_endeavour and CGI_RRA based on the Cell Cycle expression and the MIPS network.** The x-axis shows the selected 18 GO function terms and the y-axis indicates $-\log_{10}(p-value)$ from the different methods. Higher value corresponds to better performance.

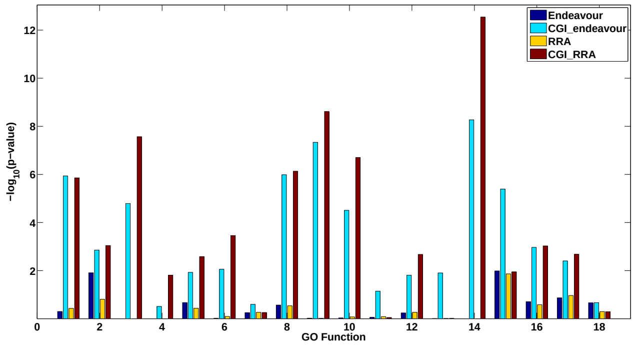

Figure S25: **Comparison of performance of Endeavour, CGI_Endeavour, RRA and CGI_RRA on predicting pathway members based on TF knockout data from [Reimand *et al.*, 2010] and the DIP interactions.** The x-axis shows the selected 18 GO function terms and the y-axis indicates $-\log_{10}(p-value)$ from the different methods. Higher value corresponds to better performance.

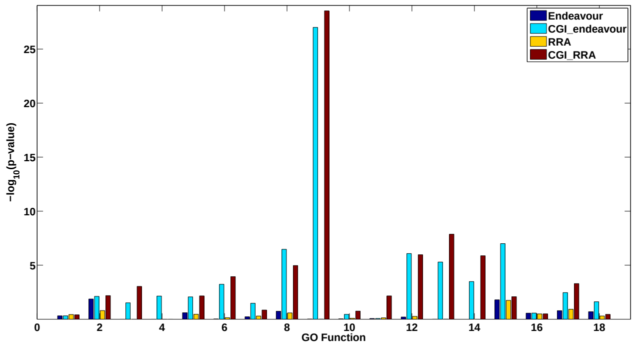

Figure S26: **Comparison of performance of Endeavour, CGI_Endeavour, RRA and CGI_RRA on predicting pathway members based on TF knockout data from [Reimand *et al.*, 2010] and the MIPS interactions.** The x-axis shows the selected 18 GO function terms and the y-axis indicates $-\log_{10}(p-value)$ from the different methods. Higher value corresponds to better performance.

Table S1. Selected optimal parameters for CGI_Endeavour

		biogrid		DIP		MIPS	
		tau	lamda	tau	lamda	tau	lamda
	ATPase_activity	0.05	0.01	0.2	0.1	0.09	0.07
	DNA_dependent_transcription_elongation	0.06	0.03	0.9	0.2	0.1	0.9
	DNA_repair	0.09	0.05	0.4	0.5	0.1	0.3
	DNA_replication	0.03	0.07	0.01	0.01	0.01	0.01
	histone_binding	0.05	0.01	0.6	0.9	0.7	0.9
	histone_modification	0.04	0.09	0.9	0.1	0.6	0.9
compendium	invasive_growth_in_response_to_glucose_limitation	0.3	0.1	0.01	0.08	0.01	0.01
	meiotic_cell_cycle	0.02	0.05	0.1	0.3	0.2	0.3
	mitotic_cell_cycle	0.07	0.01	0.07	0.09	0.7	0.2
	mRNA_processing	0.1	0.9	0.3	0.2	0.2	0.5
	protein_acylation	0.2	0.9	0.9	0.3	0.01	0.01
	regulation_of_DNA_metabolic_process	0.01	0.01	0.1	0.1	0.01	0.01
	regulation_of_organelle_organization	0.07	0.03	0.1	0.3	0.9	0.1
	response_to_DNA_damage_stimulus	0.09	0.01	0.9	0.1	0.3	0.1
	response_to_heat	0.08	0.04	0.2	0.4	0.01	0.01
	RNA_catabolic_process	0.1	0.9	0.9	0.1	0.9	0.9
	transcription_factor_binding	0.07	0.04	0.2	0.7	0.9	0.2
	transcription_from_RNA_polymerase_I_promoter	0.2	0.9	0.9	0.7	0.01	0.05
	ATPase_activity	0.4	0.1	0.9	0.9	0.01	0.01
	DNA_dependent_transcription_elongation	0.08	0.01	0.3	0.1	0.01	0.03
	DNA_repair	0.05	0.01	0.2	0.1	0.1	0.3
	DNA_replication	0.1	0.9	0.1	0.2	0.6	0.1
	histone_binding	0.04	0.01	0.01	0.08	0.01	0.03
	histone_modification	0.04	0.01	0.9	0.1	0.4	0.1
stress	invasive_growth_in_response_to_glucose_limitation	0.01	0.01	0.02	0.01	0.05	0.01
	meiotic_cell_cycle	0.09	0.01	0.1	0.1	0.4	0.1
	mitotic_cell_cycle	0.02	0.07	0.06	0.06	0.5	0.2
	mRNA_processing	0.06	0.06	0.3	0.5	0.3	0.4
	protein_acylation	0.03	0.09	0.01	0.01	0.01	0.01
	regulation_of_DNA_metabolic_process	0.02	0.03	0.09	0.09	0.5	0.2
	regulation_of_organelle_organization	0.04	0.09	0.1	0.2	0.7	0.1
	response_to_DNA_damage_stimulus	0.09	0.01	0.9	0.2	0.3	0.1
	response_to_heat	0.1	0.6	0.05	0.09	0.01	0.02
	RNA_catabolic_process	0.07	0.06	0.9	0.1	0.2	0.9
	transcription_factor_binding	0.01	0.01	0.1	0.4	0.01	0.05
	transcription_from_RNA_polymerase_I_promoter	0.1	0.4	0.02	0.09	0.02	0.01

	ATPase_activity	0.1	0.8	0.2	0.3	0.1	0.2
	DNA_dependent_transcription_elongation	0.1	0.4	0.5	0.2	0.1	0.8
	DNA_repair	0.05	0.09	0.3	0.7	0.1	0.9
	DNA_replication	0.03	0.02	0.01	0.09	0.01	0.02
	histone_binding	0.01	0.01	0.03	0.07	0.03	0.08
	histone_modification	0.03	0.01	0.09	0.02	0.04	0.08
	invasive_growth_in_response_to_glucose_limitation	0.06	0.01	0.5	0.1	0.3	0.6
Cycle	meiotic_cell_cycle	0.05	0.06	0.2	0.9	0.5	0.1
	mitotic_cell_cycle	0.1	0.9	0.07	0.09	0.4	0.9
	mRNA_processing	0.1	0.9	0.2	0.9	0.5	0.9
	protein_acylation	0.07	0.08	0.9	0.9	0.9	0.9
	regulation_of_DNA_metabolic_process	0.02	0.05	0.2	0.1	0.9	0.9
	regulation_of_organelle_organization	0.07	0.07	0.1	0.7	0.9	0.9
	response_to_DNA_damage_stimulus	0.1	0.4	0.8	0.1	0.2	0.1
	response_to_heat	0.1	0.9	0.09	0.09	0.4	0.1
	RNA_catabolic_process	0.2	0.9	0.6	0.9	0.5	0.9
	transcription_factor_binding	0.7	0.9	0.5	0.1	0.8	0.4
	transcription_from_RNA_polymerase_I_promoter	0.03	0.09	0.3	0.9	0.01	0.02

Table S2. Selected optimal parameters for CGI_RRA.

		biogrid		DIP		MIPS	
		tau	lamda	tau	lamda	tau	lamda
	ATPase_activity	0.04	0.09	0.2	0.9	0.1	0.2
	DNA_dependent_transcription_elongation	0.07	0.04	0.9	0.2	0.2	0.7
	DNA_repair	0.05	0.04	0.4	0.1	0.1	0.1
	DNA_replication	0.02	0.03	0.01	0.01	0.06	0.01
	histone_binding	0.05	0.01	0.6	0.4	0.6	0.9
	histone_modification	0.04	0.09	0.9	0.4	0.6	0.8
	invasive_growth_in_response_to_glucose_limitation	0.3	0.1	0.08	0.06	0.01	0.09
compendium	meiotic_cell_cycle	0.01	0.09	0.1	0.2	0.2	0.1
	mitotic_cell_cycle	0.06	0.01	0.08	0.09	0.9	0.2
	mRNA_processing	0.1	0.9	0.3	0.6	0.4	0.3
	protein_acylation	0.2	0.3	0.03	0.01	0.01	0.01
	regulation_of_DNA_metabolic_process	0.2	0.3	0.2	0.1	0.9	0.9
	regulation_of_organelle_organization	0.07	0.03	0.1	0.2	0.9	0.1
	response_to_DNA_damage_stimulus	0.09	0.04	0.09	0.07	0.2	0.1
	response_to_heat	0.09	0.02	0.6	0.5	0.01	0.01
	RNA_catabolic_process	0.1	0.9	0.9	0.6	0.9	0.9
	transcription_factor_binding	0.08	0.07	0.4	0.6	0.9	0.2
	transcription_from_RNA_polymerase_I_promoter	0.1	0.9	0.9	0.9	0.05	0.08
	ATPase_activity	0.09	0.01	0.9	0.8	0.01	0.04
	DNA_dependent_transcription_elongation	0.08	0.01	0.3	0.1	0.01	0.01
	DNA_repair	0.09	0.01	0.2	0.1	0.1	0.4
	DNA_replication	0.03	0.02	0.09	0.08	0.01	0.01
	generation_of_precursor_metabolites_and_energy	0.05	0.01	0.02	0.08	0.02	0.01
	histone_binding	0.01	0.05	0.9	0.1	0.2	0.4
	histone_modification	0.03	0.01	0.02	0.09	0.09	0.02
	invasive_growth_in_response_to_glucose_limitation	0.01	0.01	0.9	0.1	0.3	0.1
stress	meiotic_cell_cycle	0.09	0.01	0.06	0.04	0.6	0.1
	mitotic_cell_cycle	0.09	0.01	0.3	0.5	0.3	0.2
	protein_acylation	0.02	0.09	0.9	0.1	0.5	0.2
	regulation_of_DNA_metabolic_process	0.01	0.09	0.08	0.09	0.4	0.1
	regulation_of_organelle_organization	0.05	0.09	0.1	0.1	0.6	0.1
	response_to_DNA_damage_stimulus	0.09	0.01	0.7	0.1	0.2	0.1

	response_to_heat	0.1	0.5	0.06	0.08	0.02	0.09
	RNA_catabolic_process	0.06	0.06	0.9	0.1	0.9	0.5
	transcription_factor_binding	0.01	0.05	0.1	0.4	0.02	0.06
	transcription_from_RNA_polymerase_I_promoter	0.05	0.03	0.1	0.8	0.01	0.01
	ATPase_activity	0.1	0.8	0.1	0.6	0.1	0.1
	DNA_dependent_transcription_elongation	0.1	0.3	0.9	0.5	0.9	0.4
	DNA_repair	0.06	0.09	0.4	0.8	0.09	0.06
	DNA_replication	0.03	0.01	0.04	0.07	0.01	0.01
	histone_binding	0.03	0.01	0.02	0.09	0.03	0.08
	histone_modification	0.03	0.01	0.9	0.2	0.3	0.4
	invasive_growth_in_response_to_glucose_limitation	0.2	0.9	0.1	0.1	0.3	0.6
	meiotic_cell_cycle	0.05	0.09	0.8	0.3	0.2	0.9
	mitotic_cell_cycle	0.05	0.09	0.06	0.09	0.9	0.1
Cycle	mRNA_processing	0.1	0.9	0.2	0.8	0.3	0.9
	protein_acylation	0.1	0.1	0.02	0.01	0.09	0.09
	regulation_of_DNA_metabolic_process	0.02	0.03	0.2	0.1	0.9	0.1
	regulation_of_organelle_organization	0.07	0.01	0.1	0.5	0.4	0.6
	response_to_DNA_damage_stimulus	0.1	0.4	0.6	0.1	0.1	0.1
	response_to_heat	0.1	0.8	0.09	0.09	0.1	0.4
	RNA_catabolic_process	0.07	0.04	0.9	0.7	0.06	0.08
	transcription_factor_binding	0.8	0.9	0.6	0.1	0.9	0.7
	transcription_from_RNA_polymerase_I_promoter	0.03	0.09	0.1	0.5	0.02	0.01
